# Evaluation of mucociliary clearance among women using biomass and clean fuel in a periurban area of Chennai: A preliminary study

**DOI:** 10.4103/0970-2113.76298

**Published:** 2011

**Authors:** Johnson Priscilla, Ramaswamy Padmavathi, Santu Ghosh, Preetha Paul, Sitalakshmi Ramadoss, Kalpana Balakrishnan, Vijayalakshmi Thanasekaraan, A. S. Subhashini

**Affiliations:** *Department of Physiology, Sri Ramachandra University, Chennai, India*; 1 *Department of Environmental Health Engineering, Sri Ramachandra University, Chennai, India*; 2 *Department of Chest Medicine, Sri Ramachandra University, Chennai, India*

**Keywords:** Biomass fuel users, nasal mucociliary clearance, saccharin test

## Abstract

**Background:**

Nasal mucociliary clearance (NMC) plays a crucial role in the defense of the airways against inhaled substances and is affected by various factors. The effect of particulate matter on NMC in women using biomass fuel has not been well studied.

**Aim:**

This cross-sectional study was conducted to assess the NMC time in biomass fuel users and compare it with that of clean fuel users.

**Materials and Methods:**

NMC time and Peak Expiratory Flow Rate (PEFR) were determined in women of age ranging from 18 to 45 years using biomass fuel (*n*=30) and clean fuel (*n*=30). The time taken to perceive the sweet taste, following placement of saccharin 1 cm behind the anterior end of inferior turbinate was recorded as NMC time. PEFR was measured using mini-Wright peak flow meter. Comparison between groups was analyzed using *t*-test and ANOVA in *R* statistical software.

**Results:**

NMC time was significantly prolonged in biomass fuel users (765.8 ± 378.16 s) in comparison to clean fuel users (545.4 ± 215.55 s). PEFR was significantly reduced (319.3 l/min) in biomass fuel users compared to clean fuel users (371.7 l/min). Women from lower socioeconomic status, lower literacy status, older undernourished women and women cooking for>15 years had prolonged Saccharin Transit Time (STT) and reduced PEFR.

**Conclusions:**

This study highlights the effects of indoor air pollution on respiratory defense mechanism. This simple noninvasive, inexpensive, screening test can be used as an early indicator of respiratory damage caused by exposure to air pollutants.

## INTRODUCTION

Indoor air pollution is one of the environmental risk factor affecting mainly the rural population of developing countries. Comparative risk assessment by WHO attributes 1.6 million premature deaths annually to indoor smoke in developing countries.[1] The range of effects due to exposure to particulate matter due to combustion of biofuels is broad, affecting the respiratory and cardiovascular systems and extending to children and adults and to a number of large, susceptible groups within the general population.[2]

The mucociliary escalator is the primary defense mechanism against inhaled particulate matter. Mucociliary clearance comprises of the cephalad movement of mucus caused by the cilia lining the conducting airways until it can be swallowed or expectorated thereby protecting the human upper and lower airways from deleterious effects of inhaled pollutants, allergens, and pathogens.[3] The nasal mucociliary clearance (NMC) system transports the mucus layer that covers the nasal epithelium toward the nasopharynx by ciliary beating at a frequency of 7–16 Hz at body temperature and is controlled by certain physiological, anatomic, and biochemical variables.[4] Physiological factors such as age, sex, posture, sleep, exercise, temperature (<10 °C and >45°C) also influence the duration of NMC. When disruption of NMC occurs, respiratory secretions accumulate and impair pulmonary function, reduce lung defenses, and increase the risk for infection.[5] Tobacco smoke and environmental pollution due to combustion of biomass fuel are suspected to have a depressant effect on NMC and may lead to the development of various respiratory diseases. This also depends on factors such as pollutant concentration and the duration of exposure.[6] The underlying pathophysiology is stasis of sinonasal secretions due to ineffective sinonasal mucociliary clearance followed by subsequent bacterial overgrowth, frank infection, and/or inflammation. Nasal mucociliary clearance, the mirror image of bronchial mucociliary clearance is thus a biomarker of nasal mucosal function.[7]

Limited information is available regarding the effect of biomass fuel smoke on NMC in our country. Moreover, understanding the effect of biomass fuel smoke on NMC may advance our understanding of pathogenesis of chronic effects of such exposures. The mucociliary clearance of tracheobronchial tree can be assessed using bronchial spray mixed with radioactive compound and following with gamma camera, but it is a costly and cumbersome procedure and is not suited for field studies. Hence, this simple noninvasive method was chosen for this cross-sectional study to evaluate the mucociliary clearance in apparently healthy women dwelling in a periurban area using biomass fuel and clean fuel for cooking.

## MATERIALS AND METHODS

This cross-sectional study was conducted in 30 apparently healthy biomass fuel users (life time users of wood, dung cake, crop residues) and 30 clean fuel users (life time liquefied petroleum gas users). Women with history of cooking exposure to either biomass fuel alone or clean fuel alone for a minimum period of 2 years were included in the study. These female subjects of age group ranging from 18 to 45 years were randomly selected from a periurban area in Chennai. Informed written consent was obtained from all the study subjects.

Details regarding cooking fuel, duration of exposure were collected using a pretested validated household questionnaire. Multiple fuel users (*n*=18), smokers/passive smokers (*n*=15), and tobacco chewers/snuff users (*n*=7), were excluded. A complete ear, nose, and throat examination was also performed to rule out diseases (sinusitis, nasal polyps, allergic rhinitis, and deviated nasal septum), which are known to affect the mucociliary clearance. Thereby, women with history of (h/o) deviated nasal septum/nasal polyp (*n*=2), allergic disorders (*n*=3), h/o intake of any medications particularly antihistaminics (*n*=2), respiratory or nasal symptoms within the preceding 2 weeks (*n*=2) were also excluded. Moreover, self-reported diabetes (*n*=3), and other factors such as primary ciliary dyskinesia, bronchiectasis, valvular heart diseases, bleeding diatheses, h/o exposure to formaldehyde, ammonia, phenols were also excluded from the study. Ten women refused to participate in the study in spite of their eligibility. Thus, 122 women were contacted in order to reach the desired sample size of 60 study subjects.

The nasal mucociliary clearance was studied using the saccharine method of Anderson *et al*.[8] A 1 mm particle of saccharine was placed on the floor of the nose, just behind the anterior end of the inferior turbinate and the test was carried out in sitting position with head flexed about 10° to avoid particle falling backwards and the time required by the subject to perceive the sweet taste was noted. The test was carried out on both nostrils with an interval of half an hour. The time of mucociliary clearance of each nostril was noted separately. Nasal mucociliary clearance time is the average time of the mucosal clearance of the two nostrils. The subjects were advised to avoid nasal manipulation, sniff, cough, inhale or exhale forcefully during the test, and were simply told to report any change in taste. Subjects were blinded about the nature of particle. (The subjects were informed that some harmless edible particle will be placed in the nostril and they were not informed about its nature.) A single examiner performed the test in all subjects to avoid inter observer variability. Peak Expiratory Flow Rate (PEFR) was recorded using the Wright’s Peak flow meter. At least three readings were taken and the maximal value was noted down.

Access template was used for data entry and the analysis was performed using *R* statistical software version 2.8.1. Saccharin Transit Time (STT) and PEFR are expressed in terms of mean and standard deviation. Comparisons between the groups were analyzed by *t*-test and ANOVA and the significance was taken at 0.05 level.

## RESULTS

**Figure 1:**
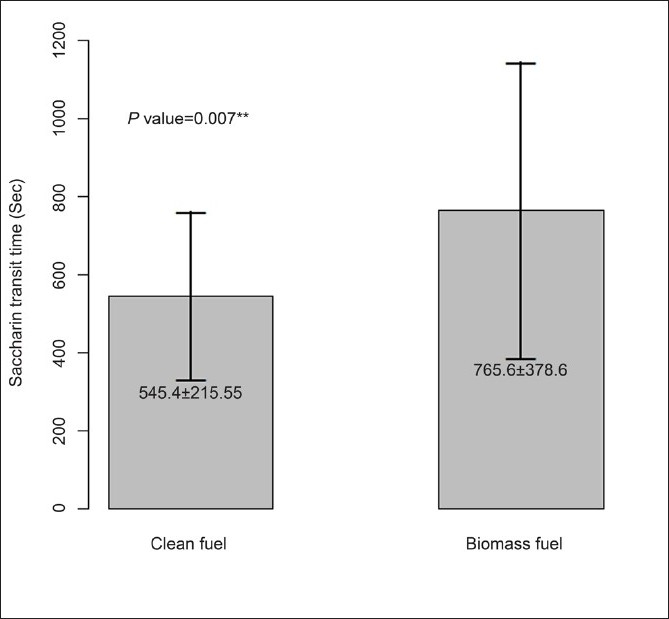
Comparison of STT between clean fuel and biomass fuel using women

**Figure 2:**
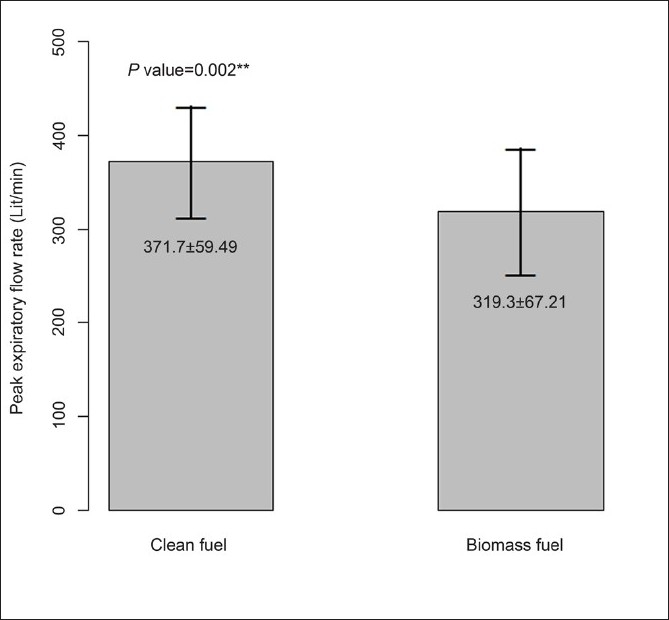
Comparison of PEFR between clean and biomass fuel using women

This study has compared NMC and PEFR between 30 clean fuel using women and 30 biomass fuel using women. Both STT and PEFR were considered as the outcome variables. The descriptive characteristics of the study population is given in Table 1. NMC time was significantly (*P* = 0.007) prolonged in biomass fuel users (765.8 ± 378.16 s) in comparison to clean fuel users (545.4 ± 215.55 s) Figure 1. PEFR was significantly (*P* = 0.002) reduced in biomass fuel users (319.3 ± 67.21 l/min) when compared with LPG users (371.7 ± 59.49 l/min) [Figure 2]. In addition, both STT and PEFR were also compared across the several subcategories as shown in Table 1. The prolongation of STT increased with increasing years of exposure (*r* = 0.47). NMC time was significantly prolonged in illiterates (*P* = 0.014), and the PEFR was also significantly reduced in this group (*P* = 0.000). Women from lower socioeconomic status (total family income <Rs. 25 000 per annum) and lower literacy status (not able to read and write), women dwelling in kutcha houses, older women (40–50 years), undernourished women (BMI<18), and women cooking for >15 years had prolonged STT and reduced PEFR.

**Table 1:** Comparison of STT and PEFR among different age, BMI, demographic and other sources of particulate matter categories of the study group

Study variables		N	STT (s)	*P* value	PEFR (l/min)	*P* value
Age	(20–29)	28	625.6 ± 352.00	0.586	363.9 ± 60.45	0.134
	(30–39)	17	638.6 ± 237.99		334.1 ± 68.65	
	(40–50)	15	731 ± 364.09		324 ± 76.51	
Income	≥25000	54	647.3 ± 339.8	0.556	351.5 ± 66.6*	0.041
	<25000	6	730.5 ± 115.07		291.7 ± 63.7	
BMI	≥18	50	651.9 ± 323.93	0.844	348.2 ± 71.08	0.497
	<18	10	674.3 ± 345.98		332 ± 52.66	
House type	Pucca	20	586.6 ± 189.29	0.247	367.5 ± 69.2	0.077
	Kutcha	40	690.1 ± 372.08		334.5 ± 65.87	
Kitchen type	Indoor	44	601.1 ± 258.61	0.029	350.5 ± 64.62	0.35
	Outdoor	16	805.6 ± 436.39		331.9 ± 77.822	
Ventilation type	Well ventilated	52	647.3 ± 316.6	0.616	354.6 ± 66.05	0.007
	Poorly ventilated	8	709.8 ± 393.48		286.2 ± 53.16	
Education	Literate	44	594.4 ± 295.9*	0.014	365.2 ± 64.75*	0.000
	Illiterate	16	824 ± 350.1		291.2 ± 44.85	
Family size (no. of persons	1–4	37	604.2 ± 268.55	0.12	352.2 ± 70.71	0.341
at home)	5–8	23	738.4 ± 391.46		334.8 ± 64.09	
Incense usage	Never	4	725.8 ± 650.28	0.582	347.5 ± 65	0.257
	Irregular	39	623.3 ± 234.71		335.4 ± 70.74	
	Regular	17	713.4 ± 414.12		368.2 ± 60.75	
Mosquito coil usage	Never	14	605.9 ±218.89	0.129	365±72.93	0.431
	Irregular	27	747.6 ± 334.03		335.6 ± 62.47	
	Regular	19	561.6 ± 355		345.3± 73.06	

Data expressed as Mean ± SD

* *P* < 0.05; STT: Saccharin transit time; PEFR: Peak expiratory flow rate

## DISCUSSION

This cross-sectional study has evaluated both NMC and PEFR in nonsmoking women using biomass fuel and compared it with those women using clean fuel for cooking. PEFR, a lung function parameter and NMC were significantly altered in women using biomass fuel. The significantly prolonged STT in biomass fuel users has highlighted the deleterious effects of biomass fuel usage on respiratory system. Tobacco smoke is known to cause depression of NMC.[9] Very few studies have been conducted on effects of biomass smoke on mucociliary clearance. The prolongation of STT in these nonsmoking study subjects could be probably due to mucus abnormality and ciliary malfunction. Earlier studies also indicate that chronic exposure to biomass fuel smoke could result in structural changes of the respiratory mucosa including epithelial sloughing, intracellular edema, and mitochondrial swelling.[10] The prolongation of STT increased with increasing years of exposure. The increase in STT could also be due to the additive effect of aging. As this is a preliminary study, additional statistical analysis to assess the contribution of other risk factors such as age could not be performed due to small sample size. The STT in clean fuel users was similar to normal mucociliary clearance time as reported by other studies such as Golhar and Arora from Chandigarh in which STT was 6.2 min and Golhar from Nagpur in which it was 7.2 min.[11,12]

This study has also evaluated PEFR, a lung function parameter. Biomass users had reduced PEFR compared to women using LPG. The reduction in lung function due to exposure to environmental pollution can be due to several inflammatory processes. For example, upregulation of P-selectin expression in platelets following activation plays an important proinflammatory role in mediating interactions among neutrophils, platelets, and the vascular endothelium.[13] Studies conducted from rural India has reported increased leukocyte aggregates and increased CD11/CD18 expression on PMN and CD62P expression on platelets in women exposed to biomass fuel.[14] In addition, compromised lung is prone for repeated respiratory viral infections leading to desquamation of epithelial cells of the lung, microvascular dilation, edema, and an inflammatory cell infiltrate. The lung damage caused by exposure to particulate matter emitted by combustion of biomass fuel will predispose the respiratory tract to bacterial infection by interfering with mucociliary clearance and by reducing the bacterial killing by alveolar macrophages.[15] Thus, the significant reduction in PEFR in biomass fuel users could be attributed to the toxic effects of the components of smoke, free radicals,[16,17] acute neutrophilic airway inflammation.[18] A decline in pulmonary functions was also observed in our earlier study conducted in biomass fuel users in Tamil Nadu.[19]

This study has highlighted the effects of biomass fuel exposure in apparently healthy adult women in whom STT has been used as an indicator for assessing the functional status of NMC. The early effects of biomass on lung function indicates the need for intervention in this vulnerable category as chronic exposure can lead to development of Chronic Obstructive Pulmonary Disease (COPD), asthma and lung cancer, increasing the morbidity and mortality in women. Large scale studies and experimental studies are required in this area of research to establish the pathogenesis of the lung damage caused by exposure to biomass fuel. STT can be used as an economical biomarker in large scale epidemiological studies in developing countries where resources for research are limited.

In conclusion, this preliminary study has shown that the NMC time is prolonged in women using biomass fuel. PEFR is reduced significantly in biomass fuel using women. This study has shown the effects of indoor air pollution on respiratory defense mechanism. This study highlights the importance of this simple non invasive, inexpensive, screening test that can be used as an early indicator of respiratory damage caused by exposure to air pollutants. In addition, dissemination of the findings of this study to the general public and the health officials regarding the rising burden of indoor air pollution and its respiratory health effects will pave way in creating awareness among the young and middle aged women about the implications of indoor air pollution so that proper intervention strategies can be implemented in order to reduce the morbidity and mortality.
